# Toxicological Responses of Photosynthetic Genes in *Chlorella vulgaris* Exposed to Environmentally Relevant Concentrations of TiO_2_ Nanoparticles

**DOI:** 10.3390/ijms262110271

**Published:** 2025-10-22

**Authors:** Gester G. Gutiérrez, Fernando Rivas-Valdés, Bárbara P. Benavente, René Olivares, Matías I. Hepp, Ricardo O. Barra, Roberto Urrutia

**Affiliations:** 1 Faculty of Environmental Sciences and EULA Center, Universidad de Concepción, Concepción 4030000, Chilericbarra@udec.cl (R.O.B.); rurrutia@udec.cl (R.U.); 2 Department of Environmental Chemistry, Faculty of Sciences, Universidad Católica de la Santísima Concepción, Concepción 4030000, Chile; 3 Biomedical Sciences Research Laboratory, Department of Basic Sciences and Morphology, Faculty of Medicine, Universidad Católica de la Santísima Concepción, Concepción 4090541, Chilemhepp@ucsc.cl (M.I.H.); 4 Faculty of Medicine and Science, Universidad San Sebastián, Concepción 4030000, Chile; 5 Aquaculture Biotechnology and Genomics, Department of Oceanography, Universidad de Concepción, Concepción 4030000, Chile

**Keywords:** titanium dioxide nanoparticles, *Chlorella vulgaris*, photosynthesis, gene expression, biomarkers, hormesis, aquatic ecotoxicology

## Abstract

Engineered nanoparticles are increasingly released into aquatic environments, raising concerns about their effects on primary producers. Titanium dioxide nanoparticles (TiO_2_ NPs), one of the most widely used nanomaterials, are frequently detected at low concentrations in surface waters. Here, we investigated the impact of environmentally relevant TiO_2_ NP concentrations (1.1–17.6 µg/L) on the freshwater microalga *Chlorella vulgaris* by combining standardized growth inhibition bioassays with transcriptional analysis of photosynthesis-related genes. Cultures were exposed for 72 h following OECD TG 201, and cell density, growth factor (GF), and specific growth rate (µ) were determined to validate bioassay reliability. Gene expression of six photosynthetic genes (*atpB*, *psaA*, *psaB*, *psaD*, *psbA*, and *rbcL*) was quantified by RT-qPCR and normalized against 18S rRNA. Statistical analyses included Shapiro–Wilk and Levene’s tests, followed by one-way ANOVA with Bonferroni or Dunnett T3 post hoc corrections. The results showed a hormetic growth response, with stimulation at intermediate NP concentrations and no inhibition at the highest dose. At the molecular level, *rbcL* was significantly repressed at 1.1–4.4 µg/L, while *psaA* and *psaD* were upregulated at 8.8–17.6 µg/L, indicating compensatory reinforcement of photosystem I. These divergent transcriptional trajectories demonstrate that molecular endpoints reveal sublethal effects not evident from cell counts alone. Overall, this study highlights the potential of photosynthesis-related genes as early biomarkers for detecting nanoparticle-induced stress in aquatic primary producers.

## 1. Introduction

The increasing production and use of engineered nanoparticles (NPs) has led to their continuous release into aquatic environments, raising concerns about long-term ecological risks. Among them, titanium dioxide nanoparticles (TiO_2_ NPs) are among the most widely used nanomaterials and are frequently detected in natural waters due to their extensive application in industrial, cosmetic, and consumer products [1,2,3,4]. Their nanoscale size (<100 nm) enhances reactivity and photochemical activity [5,6,7,8,9,10,11,12,13], but also promotes direct interactions with cellular components, potentially disrupting essential processes such as photosynthesis and energy metabolism in aquatic organisms [14,15,16,17,18,19,20,21].

Despite the growing attention on NP pollution, important knowledge gaps remain regarding their effects at environmentally realistic concentrations. Most ecotoxicological studies have focused on the milligram-per-liter range, where acute toxicity or mortality is observed, but these exposure levels do not reflect the subtle and sublethal effects that occur at microgram-per-liter concentrations commonly found in aquatic systems [22,23,24,25,26]. Such effects in primary producers may alter productivity, food web dynamics, and ecosystem stability.

To explore these sublethal responses, we selected *Chlorella vulgaris*, a model freshwater microalga widely used in standardized algal growth inhibition tests (OECD TG 201). This species plays a key role as a primary producer, is sensitive to pollutants, and is ecologically relevant due to its central role in aquatic trophic chains. Unlike *Raphidocelis subcapitata* or *Desmodesmus subcapitata*, *C. vulgaris* possesses a robust cell wall and chloroplast structure [27,28], making it particularly suitable for evaluating nanoparticle interactions, including agglomeration and photosystem disruption.

Previous studies have shown that TiO_2_ NPs can alter chloroplast membranes, inhibit ATP and NADPH synthesis, and disrupt photosystem II (PSII), ultimately reducing photosynthetic efficiency [29]. However, these effects remain poorly characterized at environmentally relevant concentrations, particularly at the molecular level. Gene expression analysis has emerged as a sensitive tool to detect early responses to contaminants before visible toxicity occurs. Focusing on photosynthesis-related genes such as *psaA*, *psaB*, *psaD*, *psbA*, *rbcL*, and *atpB* can provide valuable insights into how nanoparticles affect energy capture, electron transport, and carbon fixation pathways [30,31,32,33]; importantly, this approach is consistent with nanotoxicology work in green microalgae where sublethal endpoints—including PSII performance and transcriptional changes—have been used to uncover early effects of metal-oxide nanoparticles [34,35,36,37,38,39].

Most ecotoxicological studies on nanoparticles in microalgae, however, have relied on single, independent endpoints such as growth inhibition, pigment content, or chlorophyll fluorescence. While informative, these approaches often overlook the complexity of cellular stress responses and fail to identify sublethal mechanisms. Recent studies demonstrate that integrative approaches provide a more realistic perspective: genome-wide molecular profiling has shown that prolonged exposure to nanomaterials can trigger adaptive responses undetectable by conventional growth assays [40]. Similarly, combined physiological and molecular analyses in *Chlorella vulgaris* exposed to silver nanoparticles revealed distinct modes of toxicity between ionic and nanoparticulate forms, highlighting the limitations of evaluating endpoints in isolation [41]. These findings underscore the importance of linking standardized bioassays with mechanistic biomarkers to improve the ecological relevance of nanoparticle risk assessment. In addition, this integrative approach is highly relevant for environmental considerations when nanoparticles are proposed as solutions in agriculture or pollutant remediation at low doses. By combining classical and molecular endpoints, such analyses provide a broader perspective on both the short- and long-term consequences of nanoparticle doping, ensuring a more balanced assessment of their potential benefits and risks [42].

Finally, hormesis—a biphasic response characterized by low-dose stimulation and high-dose inhibition—is well documented in algae exposed to chemical stressors, including nanomaterials [43]. Understanding how gene-expression patterns align with hormetic growth responses is crucial to predict potential changes in biomass production and ecosystem balance.

The present study aimed to evaluate the effects of TiO_2_ NPs at environmentally relevant concentrations (1.1–17.6 µg/L) on the growth and photosynthetic gene expression of *C. vulgaris*. Specifically, we sought to: (i) determine whether TiO_2_ NPs induce hormetic responses at the cellular level; (ii) assess the dose-dependent regulation of photosynthesis-related genes; and (iii) explore the potential of these genes as early molecular biomarkers of nanoparticle stress in aquatic primary producers.

## 2. Results

### 2.1. Standard Analysis of TiO_2_ Nanoparticles

**Figure 1:**
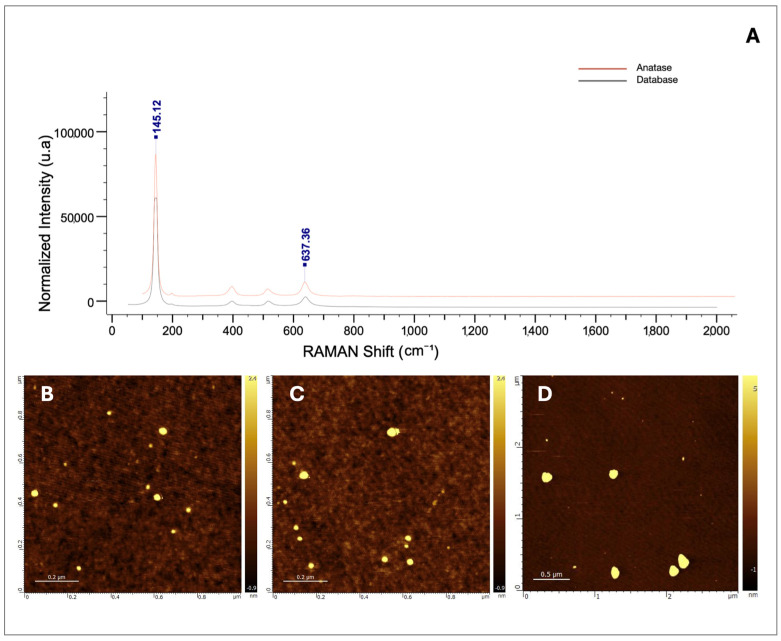
(**A**) Raman spectrum of TiO_2_ nanoparticles showing characteristic anatase peaks, confirming the crystalline phase of the material. (**B**–**D**) Atomic force microscopy (AFM) images of TiO_2_ nanoparticles dispersed in algal growth medium at stock concentrations of 10 µg/L (**B**), 50 µg/L (**C**), and 100 µg/L (**D**). AFM analysis revealed concentration-dependent agglomeration patterns, with larger aggregates observed at higher nanoparticle concentrations.

**Figure 2:**
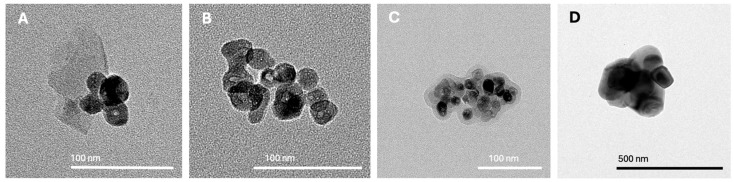
Transmission electron microscopy (TEM) images of commercial TiO_2_ nanoparticles dispersed in algal growth medium at environmentally relevant concentrations. Panels show agglomeration patterns at 1.1 µg/L (**A**), 4.4 µg/L (**B**), 8.8 µg/L (**C**), and 17.6 µg/L (**D**).

The effects of TiO_2_ nanoparticles on *Chlorella vulgaris* cultures at environmentally relevant concentrations were evaluated after confirming nanoparticle identity and purity through physicochemical characterization. Raman spectroscopy (Figure 1A) showed spectral peaks matching those of the anatase crystalline phase, with a high-quality index (HQI = 99.39) confirming a strong spectral match. Complementary AFM imaging (Figure 1B–D) revealed the surface morphology of dispersed nanoparticles, demonstrating concentration-dependent agglomeration patterns at 10, 50, and 100 µg/L. Transmission electron microscopy (TEM) (Figure 2A–D) further confirmed that primary particles were smaller than 100 nm, while agglomerates exceeded 100 nm, with aggregate size increasing proportionally to nanoparticle concentration. The predominance of anatase was clear, whereas rutile detection was less consistent due to differences in Bravais lattice structure [9,44]. Together, these results confirm the physicochemical identity of the TiO_2_ nanoparticle standard and highlight the direct implications of agglomeration dynamics for subsequent bioassay behavior.

### 2.2. Cell Inhibition Assay

The validity of the bioassay was confirmed following the criteria established in OECD Guideline 201 (2011), using growth data from *C. vulgaris* cultures maintained under controlled laboratory conditions. Key parameters—including mean (σ), standard deviation (SD), growth factor (GF), specific growth rate (µ), and coefficient of variation (CV%)—were calculated based on triplicate measurements from three independent biological replicates per treatment.

The control met all OECD 201 validity criteria, achieving a growth factor (GF) of 19.90 (threshold ≥ 16), indicating sufficient cell proliferation during the 72 h exposure period. The specific growth rate (µ) ranged from 0.89 to 1.00 d^−1^, consistent with the expected range for optimal *C. vulgaris* growth.

**Figure 3:**
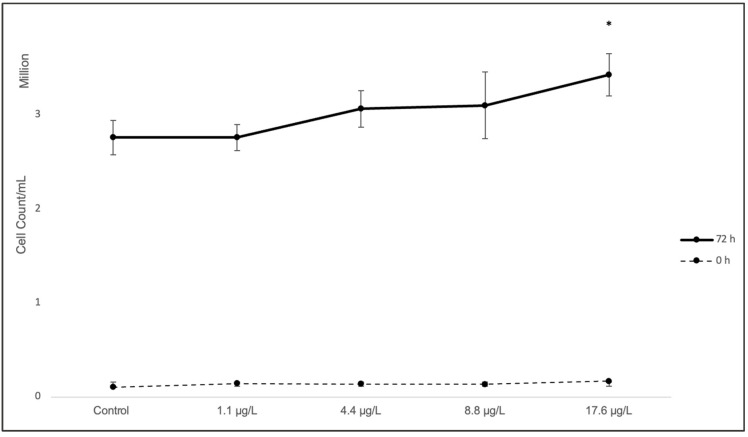
Cell density of *Chlorella vulgaris* after 72 h exposure to TiO_2_ nanoparticles (1.1–17.6 µg/L). Data are expressed as mean ± SD (n = 3 biological replicates). The dashed line indicates the initial density at 0 h, while the solid line represents the final mean density after 72 h. An asterisk indicates a significant difference compared with the control (* *p* < 0.05; one-way ANOVA, Dunnett’s post hoc test).

Assay reproducibility was evaluated through the coefficient of variation (CV%), which was 6.66% in the control (<20%), indicating low variability and high precision. All treatment groups also exhibited CV% values below 20%, confirming the reliability of the experimental setup for subsequent ecotoxicological interpretation (Figure 3).

These findings confirm that the bioassay met the validation criteria established by OECD 201 (2011), supporting the reliability of subsequent gene expression analyses conducted within this ecotoxicological framework [45]. For the *C. vulgaris* cell inhibition assay, a Dunnett’s T3 post hoc test was applied to compare the control group with the different TiO_2_ concentrations (1.1 µg/L, 4.4 µg/L, 8.8 µg/L, and 17.6 µg/L). Figure 3 shows the cell density recorded at the end of the 72 h bioassay.

The control group achieved a growth factor (GF) of 26.2 (threshold ≥ 8) and a coefficient of variation (CV%) of 6.7%, both within the acceptable range defined by OECD 201 (CV% < 20%). All treatment groups also met these criteria, confirming the validity of the bioassay for assessing ecotoxicological effects on microalgae.

The statistical analysis using Dunnett’s T3 post hoc test revealed evidence of growth stimulation in *C. vulgaris* cultures. At 72 h, comparisons between the control group and the treatments with 1.1 µg/L and 4.4 µg/L of TiO_2_ showed no statistically significant differences.

However, the comparison with 8.8 µg/L revealed a mean difference of −28.33 (*p* = 0.090), indicating a trend toward statistical significance, although not reaching the conventional threshold (*p* < 0.05). In contrast, the highest concentration (17.6 µg/L) showed a highly significant difference from the control (mean difference = −61.00; * *p* < 0.001), marking the point at which the stimulatory effect on cell proliferation becomes evident in *C. vulgaris* cultures (Table 1).

As shown in Figure 2, after 72 h of exposure, the number of cells in *C. vulgaris* cultures increased proportionally with the concentration of TiO_2_ nanoparticles. This response may be partially attributed to the formation of larger nanoparticle agglomerates at higher concentrations (Figure 1B). Such behavior has been previously reported in media containing salts or other solutes [46].

In natural aquatic environments, where dissolved solute concentrations are typically higher than under controlled laboratory conditions, the agglomeration behavior of nanomaterials may become even more unpredictable, potentially exacerbating ecological risks. This contrasts with the risks reported in studies where smaller nanoparticles exhibit higher reactivity and greater potential for internalization into photosynthetic organisms [16,47].

However, the increase in both the concentration and size of TiO_2_ nanoparticle agglomerates may also intensify ecological risk by promoting excessive microalgal biomass accumulation. This process can contribute to eutrophication, which in turn leads to trophic imbalances and deteriorated water quality, ultimately affecting biodiversity and ecosystem health.

These results indicate that TiO_2_ nanoparticles, from an environmentally relevant concentration of 17.6 µg/L, trigger a hormetic response in *C. vulgaris* cell density. This biphasic effect reinforces the selection of this concentration range for evaluating early molecular responses through gene expression analysis. Detecting such sublethal biochemical signals is essential for strengthening ecotoxicological assessments of nanomaterials, as they may not cause acute toxicity but still disrupt cellular metabolism.

### 2.3. Standardization of Primers

For the gene expression analyses, first, the set of primers synthesised for the *18S*, *atpB*, *psaA*, *psaB*, *psaD*, *psbA*, and *rbcL* genes was standardised. The corresponding standard curves were then performed to verify the efficiency and linearity of the primers, as shown in Table 2.

Efficiency values were within the acceptable range of 90–110%, supporting reproducible amplification for the *psaB* and *psbA* genes. These targets demonstrated optimal efficiency and consistent amplification, validating their suitability for relative quantification. In contrast, the *psaA* gene showed an efficiency slightly above 110%, suggesting possible overamplification, while *atpB* also exceeded the ideal threshold. Therefore, future analyses should consider optimizing the reaction conditions for *psaA* and *atpB* to improve assay performance.

### 2.4. Gene Expression

Prior to the ANOVA, Levene’s test was applied to evaluate the assumption of homogeneity of variances across treatments for each target gene. The test revealed that variances were homogeneous for *atpB*, *rbcL*, and *psbA* (*p* > 0.05), whereas significant heterogeneity was detected for *psaA*, *psaB*, and *psaD* (*p* < 0.05). In all cases, the subsequent ANOVA remained valid because corrections were applied through Bonferroni and Dunnett T3 post hoc procedures when necessary (Table 3).

The one-way ANOVA showed significant treatment effects for five out of six genes examined (*p* < 0.05). Only psbA failed to show statistically significant variation among treatments (*p* = 0.748) and was therefore excluded from further analysis and discussion.

For atpB, the one-way ANOVA indicated a significant overall effect (*p* = 0.003). However, subsequent post hoc tests did not reveal significant differences between individual treatments and the control (Table 3; Appendix A Table A1). This suggests that, although a global trend was detected, the variability among replicates limited the identification of specific concentration-dependent effects.

For *rbcL*, a consistent downregulation was observed at 1.1 and 4.4 µg/L compared with the control (*p* < 0.05), indicating inhibition of carbon fixation pathways at lower nanoparticle concentrations. In contrast, *psaA* exhibited a significant upregulation at 8.8 µg/L (*p* = 0.018), while *psaD* was strongly induced at both 8.8 and 17.6 µg/L (*p* < 0.05), suggesting activation of photosystem I components under elevated nanoparticle exposure.

*psaB* also displayed significant differences at 8.8 µg/L (*p* < 0.05); however, its expression profile showed greater variability across treatments, making the overall trend less consistent compared to other genes.

In summary, significant and consistent concentration-dependent alterations were detected for *rbcL*, *psaA*, *psaD*, and *psaB*, whereas *atpB* showed an overall ANOVA effect without treatment-specific significance, and *psbA* remained unaltered across all conditions.

**Figure 4:**
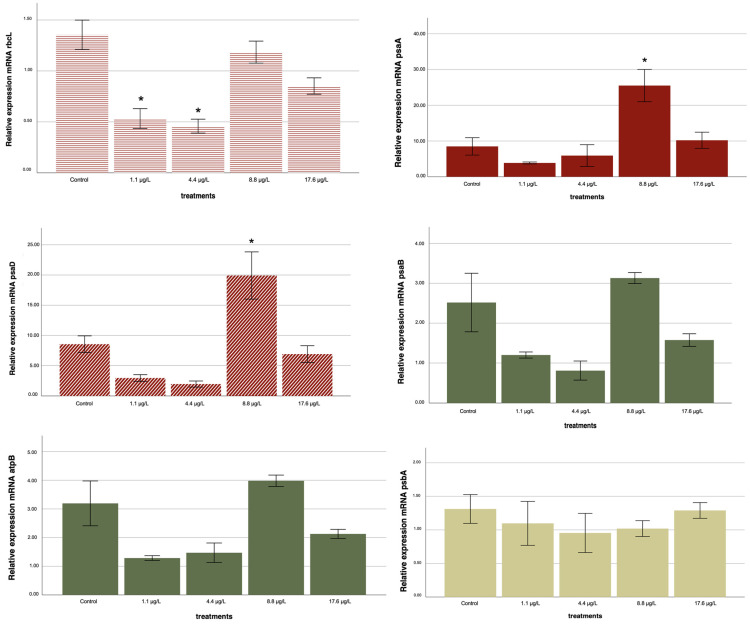
Relative mRNA expression of six photosynthesis-related genes in *C. vulgaris* after 72 h exposure to TiO_2_ nanoparticles (1.1–17.6 µg/L). Expression values were calculated using the standard curve method, normalized to 18S rRNA, and are presented as relative expression ratios. Data are shown as mean ± 1 standard deviation (n = 3 biological replicates). Asterisks indicate significant differences compared with the control (* *p* < 0.05; one-way ANOVA, Dunnett’s post hoc test). Bar colors are used only for visual grouping: red, genes showing at least one significant change; green, genes showing changes without statistical significance; and yellow, genes without detectable alterations under any treatment.

These transcriptional responses across all six genes are summarized in Figure 4, which illustrates the relative expression profiles after 72 h exposure to TiO*_2_* nanoparticles.

Taken together, these results indicate that TiO_2_ nanoparticles can elicit both repression and stimulation of photosynthesis-related genes in *C. vulgaris*, in a concentration-dependent and gene-specific manner, supporting the occurrence of hormetic-like responses at the molecular level.

The transcriptional responses of *C. vulgaris* to TiO_2_ nanoparticles were not monotonic but instead followed dose-dependent trajectories that paralleled the hormetic pattern observed in cell density. At the lowest concentrations tested (1.1 and 4.4 µg/L), *rbcL* expression was significantly repressed, indicating an early inhibition of carbon fixation. However, this repression was progressively alleviated at higher concentrations, with a partial recovery at 8.8 µg/L and a return to near-control levels at 17.6 µg/L, suggesting an acclimatory adjustment of the photosynthetic machinery. In contrast, *psaA* exhibited a pronounced induction at 8.8 µg/L, reflecting a transient upregulation of PSI reaction-center components, but this effect diminished at 17.6 µg/L, where expression remained slightly above the control but no longer statistically significant. *psaD* followed a distinct trajectory, showing strong upregulation at 8.8 µg/L and maintaining elevated expression at 17.6 µg/L, pointing to a sustained reinforcement of PSI electron-transfer capacity under stronger stress.

Together, these gene-specific patterns reveal a coordinated but divergent regulatory strategy: the repression of *rbcL* at low doses coincides with a limitation in carbon assimilation, while the induction of PSI components at intermediate and high doses likely serves to sustain electron transport and balance energy metabolism under nanoparticle stress. This molecular behavior mirrors the hormetic stimulation of cell growth observed in the bioassay, where intermediate doses promoted cell proliferation before the effect attenuated at the highest concentration. Thus, the integrated response suggests that *C. vulgaris* initially restricts carbon fixation but subsequently engages compensatory mechanisms through PSI activation, providing a mechanistic basis for the hormetic phenotype detected at the cellular level.

**Figure 5:**
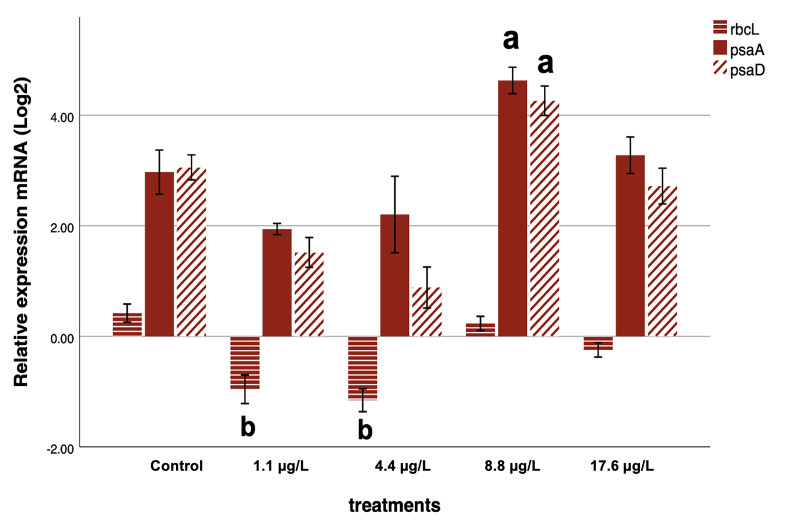
Relative mRNA expression (log_2_) of *rbcL*, *psaA* and *psaD* genes in *Chlorella vulgaris* after 72 h exposure to TiO_2_ nanoparticles (1.1–17.6 µg/L) compared to the control. Data were normalized against the 18S reference gene and are expressed as mean ± 1 standard deviation (n = 3 biological replicates). Letter a denotes significant overexpression, whereas letter b denotes significant repression of genes (*p* < 0.05; one-way ANOVA followed by Bonferroni post hoc test).

This divergent transcriptional behavior, characterized by low-dose repression of *rbcL* and the induction of *psaA* and *psaD* at intermediate to high exposures, provides molecular evidence consistent with the hormetic growth response observed in the bioassay. These patterns are illustrated in Figure 5.

## 3. Discussion

### 3.1. Adaptive Response: Positive Regulation

The induction of *psaA* and *psaD* at intermediate and high nanoparticle concentrations illustrates an adaptive molecular strategy of *C. vulgaris*. Rather than a linear dose-dependent effect, the upregulation of these photosystem I subunits reflects the organism’s capacity to reconfigure electron transport pathways under stress. PSI functions as the terminal electron acceptor in the photosynthetic chain, and its reinforcement may ensure continuity of ATP and NADPH production when other parts of the system, such as carbon fixation via *rbcL*, are compromised. This type of adjustment has been associated with enhanced cyclic electron flow around PSI, a mechanism known to contribute to photoprotection by dissipating excess energy and stabilizing the redox state of chloroplasts [33].

Notably, *psaD* maintained elevated expression even at the highest exposure, whereas *psaA* induction was transient. This divergence suggests a fine-tuned regulation in which certain PSI components are preferentially sustained to guarantee electron transfer stability. Such molecular plasticity is consistent with previous reports of algae under oxidative or nanoparticle stress, where targeted reinforcement of PSI allowed acclimation without requiring generalized upregulation across the entire photosynthetic machinery. These findings highlight that the adaptive response of *C. vulgaris* is not a uniform overexpression, but a selective reinforcement of key nodes in the electron transport network [32,48].

### 3.2. Toxic Effect: Negative Regulation

The repression of *rbcL* at 1.1 and 4.4 µg/L reveals an early toxicological impact of TiO_2_ nanoparticles on the photosynthetic machinery of *C. vulgaris*. *rbcL* encodes the large subunit of RuBisCO, the key enzyme responsible for CO_2_ fixation. Its transcriptional downregulation at low doses suggests that even environmentally relevant concentrations of nanoparticles can initially compromise carbon assimilation, limiting primary productivity. Such inhibition may result from direct oxidative stress or from indirect effects related to nanoparticle agglomeration, which alters bioavailability and cellular uptake. Although speculative in this study, previous reports indicate that smaller aggregates at lower concentrations can remain more bioavailable and interact more efficiently with cells, thereby producing stronger inhibitory effects than at higher doses, where larger agglomerates tend to form and reduce effective toxicity [49].

At higher concentrations (8.8–17.6 µg/L), *rbcL* expression returned to near-control levels, indicating an apparent acclimatory adjustment. However, whether this recovery reflects a truly sustained adaptation or a transient compensation remains uncertain. It is possible that enhanced PSI activity (*psaA* and *psaD* induction) provides a temporary stabilization of ATP and NADPH pools, allowing RuBisCO activity to be restored despite ongoing nanoparticle stress. Over longer exposures, this compensatory balance may collapse if energy demands exceed the buffering capacity of PSI. Therefore, the normalization of *rbcL* at elevated concentrations should be interpreted cautiously: it may represent either genuine resilience of algal metabolism or a short-lived adjustment preceding eventual decline [29].

### 3.3. Hormetic Observations: Cell Density and Gene Regulation

The transcriptional dynamics observed in this study provide a mechanistic basis for the hormetic growth pattern detected in *C. vulgaris* cultures exposed to TiO_2_ nanoparticles. At low concentrations (1.1–4.4 µg/L), repression of *rbcL* reflects an early limitation of carbon fixation, consistent with the absence of growth stimulation in the cell-count bioassay. This initial inhibition may be linked to the higher bioavailability of small nanoparticle aggregates at low concentrations, which could penetrate cells more effectively and disrupt photosynthetic metabolism.

At 8.8 µg/L, however, the strong induction of *psaA* and *psaD* coincided with the hormetic stimulation of cell density, suggesting that reinforcement of PSI electron transport provided the energetic compensation required to overcome the temporary bottleneck in carbon assimilation. This transcriptional reconfiguration allowed cells to sustain ATP/NADPH production and redirect energy flow, supporting enhanced proliferation at intermediate exposure levels.

At 17.6 µg/L, the response diverged: *psaD* remained strongly induced while *psaA* declined, and *rbcL* normalized. This pattern corresponds to the attenuation of hormesis observed at the cellular level, indicating that while compensatory mechanisms persist, they may represent a precarious balance rather than a stable adaptation. The persistence of elevated *psaD* expression at the highest concentration suggests a prioritization of PSI stability under stress, but whether this reflects long-term acclimation or merely a transient adjustment remains uncertain.

Altogether, these results align with the conceptual framework of hormesis, where low-dose inhibition is followed by compensatory stimulation at intermediate levels and attenuation under stronger stress. The molecular data presented here provide direct evidence that the hormetic phenotype in microalgal growth emerges from the interplay between early repression of carbon fixation and compensatory induction of PSI genes [43,50].

It should be noted that the mechanistic links proposed between transcriptional changes (*psaA, psaD, rbcL*) and the hormetic growth response remain correlative in nature. While our data reveal consistent expression patterns that align with growth dynamics, no direct measurements of photosynthetic performance (e.g., Fv/Fm, electron transport rate), enzyme activity (RuBisCO), or ATP/NADPH levels were performed. Therefore, the interpretation presented here should be regarded as a proposed model integrating gene expression signals with cellular outcomes. Similar observations have been reported in the recent literature, where hormetic responses to nanomaterials were linked to differential gene expression but emphasized as correlative rather than causal, highlighting the need for integrative studies that combine molecular and physiological endpoints [51]. Future work in this direction will be essential to validate and refine the mechanistic framework proposed here.

### 3.4. Ecological Implications: A Balance Between Adaptation and Risk

The molecular and cellular responses described here also have implications for environmental risk assessment (ERA) of nanoparticles in aquatic systems. Traditional ERA endpoints, such as growth inhibition measured by cell counts, may underestimate risk when hormesis occurs. In the present study, cell density indicated stimulation at intermediate TiO_2_ concentrations, which could be misinterpreted as a neutral or even beneficial effect if evaluated in isolation. However, the repression of *rbcL* at low doses demonstrated that key photosynthetic functions were already compromised at concentrations where no visible growth inhibition occurred. This discrepancy underscores the limitations of relying exclusively on growth-based endpoints.

By integrating gene expression biomarkers into ERA frameworks, it becomes possible to detect sublethal alterations that precede or accompany hormetic stimulation. Such early molecular signals provide a more sensitive and mechanistic basis for predicting ecological consequences, particularly for primary producers that underpin aquatic food webs. Without these molecular insights, risk assessments could overlook subtle disruptions in carbon assimilation pathways, leading to contradictory conclusions: apparent growth stimulation on the one hand, but impaired photosynthetic capacity on the other.

Although cultures were continuously agitated to minimize nanoparticle sedimentation, it should be noted that agglomeration was not directly measured during the bioassays. Instead, TEM and AFM analyses performed on TiO_2_ suspensions in algal growth medium demonstrated concentration-dependent agglomeration, providing indirect evidence to contextualize the observed biological responses. Based on these findings, we infer that at lower concentrations, nanoparticles remain more dispersed, increasing their surface area and interaction potential with algal cells, which may account for the stronger inhibitory effects detected under these conditions. In contrast, at higher concentrations, the formation of larger aggregates likely reduces effective bioavailability by decreasing surface area and favoring sedimentation of clusters, thereby attenuating cellular responses. This interpretation is consistent with previous studies reporting similar dynamics in algal systems [49,52,53,54]. Such considerations highlight the importance of combining particle characterization with biological endpoints to strengthen mechanistic interpretations in nanoparticle risk assessment.

Ultimately, the combined use of classical bioassays and molecular biomarkers offers a more comprehensive evaluation of nanoparticle impacts. In the case of TiO_2_, this integrative approach reveals that the hormetic growth response is not simply benign, but reflects an underlying physiological stress that could compromise ecosystem functioning under long-term or community-level exposure scenarios [55,56].

**Figure 6:**
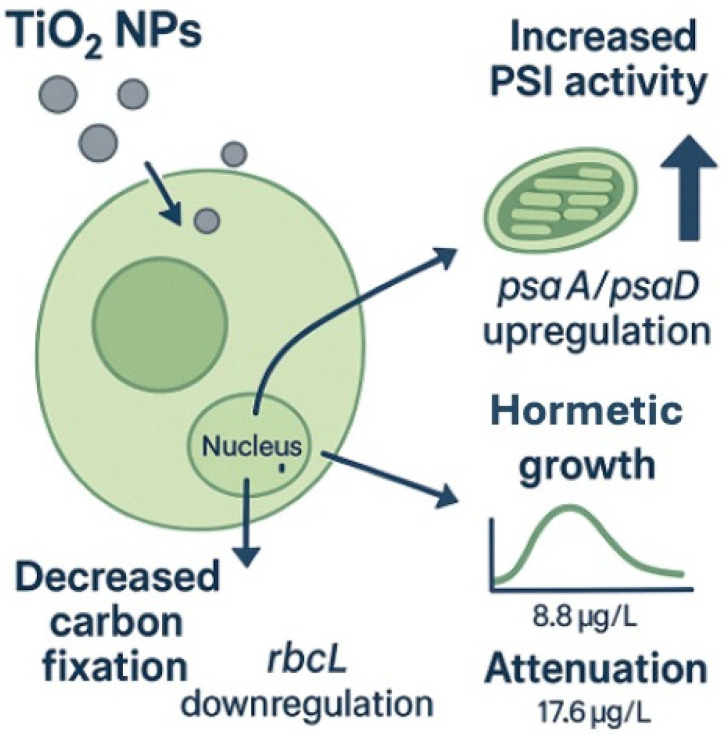
Conceptual scheme summarizing the molecular and cellular responses of *Chlorella vulgaris* exposed to TiO_2_ nanoparticles. The downregulation of rbcL at low concentrations indicates an early impairment of carbon fixation, whereas the upregulation of psaA and psaD reflects the reinforcement of PSI electron transport under stress. These transcriptional adjustments explain the hormetic growth pattern observed at 8.8 µg/L and its attenuation at 17.6 µg/L, linking gene regulation with physiological and ecological outcomes.

To integrate these findings, a conceptual scheme was developed to summarize the molecular and cellular responses of *Chlorella vulgaris* to TiO_2_ nanoparticles (Figure 6). The diagram highlights the downregulation of *rbcL* at low concentrations and the induction of *psaA* and *psaD* at intermediate and high exposures, linking these transcriptional adjustments with the hormetic growth pattern observed. This visual synthesis reinforces the interpretation that molecular regulation underlies the balance between stimulation and attenuation across exposure levels.

### 3.5. Study Limitations

One limitation of the present study is that direct quantification of TiO_2_ nanoparticle uptake by *Chlorella vulgaris* was not performed. Nevertheless, complementary TEM and AFM analyses conducted in algal growth medium at environmentally relevant concentrations provided valuable evidence of concentration-dependent agglomeration patterns, supporting the interpretation of exposure conditions. Another limitation relates to the bioassay design, since hydrodynamic parameters such as zeta potential, PDI, or DLS were not measured in the actual exposure system. These parameters could have provided additional insights into nanoparticle stability and effective exposure during the bioassay. Despite these constraints, the combined Raman, TEM, and AFM analyses ensured a robust confirmation of nanoparticle identity, morphology, and aggregation behavior under experimental conditions.

Another limitation of this study is that the amplification efficiencies of *psaA* (118%) and *psaD* (120%) exceeded the recommended range (90–110%). Although melt curve analysis and gel electrophoresis confirmed the specificity of the amplicons, such values may introduce uncertainty in relative quantification. Therefore, the results for *psaA* and *psaD* should be interpreted with caution. However, their consistency with the expression trends of other photosynthetic genes and the observed physiological responses reinforces the reliability of the conclusions.

### 3.6. Perspectives on Metabolomics and Integrated Omics Approaches

In addition to transcriptional responses, growing evidence indicates that nanoparticles can also induce significant metabolomic alterations in microalgae. TiO_2_ NPs have been reported to disrupt amino acid, lipid, and carbohydrate metabolism, leading to shifts in cellular energy balance and biochemical composition [57,58]. Such metabolomic changes often occur at sublethal concentrations and may precede visible growth effects, making them valuable early biomarkers of nanoparticle stress. Recent reviews further emphasize that integrating metabolomics with transcriptomics is essential to link gene regulation with functional metabolic outcomes and to achieve a systems-level understanding of nanoparticle impacts [59]. Although metabolomic profiling was not included in the present study, future work combining transcriptomic and metabolomic approaches will provide a more comprehensive perspective on the mechanisms of nanoparticle toxicity in primary producers.

## 4. Materials and Methods

### 4.1. Analysis of TiO_2_ Nanoparticle Standard

For toxicity testing, a stock solution was prepared using a certified Commercial TiO*_2_* nanoparticles (rutile/anatase mixture, particle size < 100 nm, ≥99.5% trace metals basis) were obtained from Aldrich (Product No. 637262, Batch No. BCCF1510), manufactured in Shanghai, China and certified by Quality Assurance, Buchs, Switzerland. dispersed in algal growth medium [45]. The physicochemical specifications, including primary particle size, crystalline phase composition, and purity, were provided by the manufacturer. Therefore, additional surface characterization techniques such as dynamic light scattering (DLS), zeta potential, or energy-dispersive X-ray spectroscopy (EDS) were not required.

To confirm particle identity and morphology, Raman spectroscopy coupled with atomic force microscopy (AFM) was performed using a Horiba LabRAM Nano 800 system (HORIBA France SAS, Loos, France). This combined technique enabled verification of the crystalline phase through Raman analysis and provided high-resolution AFM images of nanoparticle surface topography and agglomeration behavior. AFM revealed the formation of larger agglomerates at higher concentrations, offering three-dimensional insight into clustering patterns. In addition, high-resolution transmission electron microscopy (TEM; Talos F200 G2, Thermo Scientific, Waltham, MA, USA) equipped with a CMOS CETA 16M camera was used to determine particle size, morphology, and fine agglomeration structures at the nanoscale. TEM images confirmed the concentration-dependent increase in agglomerate size, complementing AFM observations and strengthening the characterization of the nanoparticle standard.

TiO_2_ NPs were selected because they are among the most widely produced nanomaterials worldwide, with extensive applications in cosmetics, paints, coatings, and food additives [60]. Due to these widespread applications, TiO_2_ NPs are frequently detected in wastewater effluents and surface waters at environmentally relevant concentrations [60,61]. Their high production volume and recurrent environmental occurrence make them a representative model contaminant for aquatic ecotoxicology.

### 4.2. Biological Analysis

The test microalga *Chlorella vulgaris* (strain CCM-UDEC 051) was obtained from the FICOLAB culture collection (Faculty of Natural and Oceanographic Sciences, Universidad de Concepción, Concepción, Chile) and maintained under standard OECD TG 201 (2011) culture conditions. *C. vulgaris* was chosen because it is widely distributed in freshwater, estuarine, and even wastewater environments, reflecting its ecological persistence and tolerance to variable conditions [62,63]. This broad occurrence highlights its ecological relevance, as it plays a central role as a primary producer in aquatic food webs. Moreover, *C. vulgaris* has been successfully used in ecotoxicological assessments under different stressors, including nutrient-rich effluents, confirming its robustness as a test organism [64]. Importantly, given its relatively resilient physiology, adverse responses in *C. vulgaris* may suggest that more sensitive phytoplankton species could be even more affected, reinforcing its protective value as an indicator in nanoparticle risk assessment.

The growth inhibition assay was performed by exposing *C. vulgaris* cultures to a series of environmentally relevant concentrations of TiO_2_ nanoparticles (mix of rutile and anatase phases). A stock suspension of 1000 μg/L was prepared, from which serial dilutions were made to obtain final exposure concentrations of 1.1, 4.4, 8.8, and 17.6 μg/L. These levels were selected based on previous environmental monitoring of the Biobío River [65], where TiO_2_ nanoparticles were detected at comparable concentrations. All treatments, including the control, were performed in triplicate (three independent biological replicates), with a culture volume of 10 mL per replicate in sterile glass vessels, ensuring adequate nutrient availability and light penetration throughout the experiment.

The exposure period was set to 72 h, following OECD TG 201 recommendations, to maintain microalgae in the exponential growth phase during the assay. Extending the exposure to longer periods (e.g., 96 h) could result in nutrient depletion and entry into stationary phase, potentially confounding growth inhibition and gene expression measurements.

Cell density and size were recorded every 24 h using an Olympus optical microscope (Olympus, Tokyo, Japan). To verify the validity of the bioassay, the specific growth rate (µ), growth factor (GF), and coefficient of variation (CV%) were then calculated as follows:

(1) $$\mu =\;\frac{\ln N2-lnN1}{t2-t1}$$

(2) $$\mathrm{GF}=\frac{Nt}{N0}$$

(3) $$\mathrm{CV}\frac{SD}{Mean}\times 100$$

where *N*0 and *Nt* represent the initial and final cell densities, respectively, and *t*1 and *t*2 are the start and end time on the measurement interval.

Once the bioassay was completed at 72 h, cell counting for cell inhibition and measurement of cell size was performed under an Olympus optical microscope. Then, the extraction of genetic material was started.

### 4.3. Obtaining RNA from Chlorella vulgaris

*Chlorella vulgaris* pellets were obtained by centrifugation at 14,000 rpm and 4 °C using a CR 22G III-HITACHI ultracentrifuge (Hitachi Koki Co., Ltd., Life-Science Instruments Division, Tokyo, Japan), completely removing the supernatant. The cell lysis process was carried out by adding 90 µL of Lysis Binding Solution Concentrate MagMAX™-96 Total RNA Isolation Kit (Qiagen, Hilden, Germany) and 10 µL of Plant RNA Isolation Aid (cat. #AM9690) to each sample, resulting in a total lysate volume of 100 µL. After pre-centrifugation at 14,000 rpm to remove cell debris, the clarified supernatant was recovered, and a 50 µL aliquot was used to continue the RNA extraction protocol according to the manufacturer’s instructions (MagMAX™-96 Total RNA Isolation Kit, Thermo Fisher Scientific, Waltham, MA, USA).

### 4.4. Primer Design

Table 4 presents the list of primers designed for the selected genes, together with their functions in cell metabolism. The primers were generated using the gene sequences available at NCBI Primer-BLAST (https://www.ncbi.nlm.nih.gov/tools/primer-blast/, accessed on 9 September 2024). For *18S* and *psaA*, primers were designed de novo in this study using consensus sequences obtained from Geneious Prime^®^ 2023.1.1, as no specific GenBank accession was available. The remaining primers were designed based on sequences retrieved from GenBank (see Table 1). The *18S* rRNA gene was selected as a reference gene and used as an internal standard for normalization of gene expression data.

Amplification efficiency and linearity were assessed by constructing standard curves for each target gene, including *atpB*, *psaA*, *psaB*, *psaD*, *psbA*, and *rbcL*, and the 18S reference gene. Analyses were performed on a QuantStudio 3 thermal cycler (Applied Biosystems) using Design & Analysis Software v2.7.0. The threshold cycle (TC) quantification method was employed, and the standard curve was generated using the Primary Analysis v1.8.0, Standard Curve v1.7.0 module. The parameters evaluated included the slope of the calibration curve (Slope), the coefficient of determination (R^2^), the amplification efficiency (Eff%), and the standard error. The sheath temperature was set at 105 °C, and a three-step melting stage analysis was applied.

### 4.5. RT-qPCR

The mRNA concentration of each sample was determined using a NanoDrop Lite Spectrophotometer (Thermo Fisher Scientific, Waltham, MA, USA) with 1 μL of extract. A total of 500 ng of RNA was reverse transcribed into cDNA following the manufacturer’s instructions for the M-MLV Reverse Transcriptase kit (Promega, Madison, WI, USA). Quantitative PCR (qPCR) was performed to determine the relative expression levels of six target genes (*atpB*, *psaA*, *psaB*, *psaD*, *psbA*, *and rbcL*) using KAPA SYBR^®^ FAST qPCR Master Mix (2X) (KAPA BIOSYSTEMS, Cape Town, South Africa) in a QuantStudio 3 thermal cycler (Applied Biosystems™, Thermo Fisher Scientific, Waltham, MA, USA).

Cycling conditions were an initial denaturation at 95 °C for 8 min, followed by 40 cycles of 95 °C for 10 s, 55 °C for 20 s, and 72 °C for 20 s, and a final melting curve stage. Each reaction was performed in triplicate (three technical replicates) for each of the three biological replicates per treatment.

### 4.6. Statistical Analysis

All statistical analyses were performed using IBM SPSS Statistics 25 (IBM Corp., Armonk, NY, USA). For bioassay data (cell density and growth inhibition), the normality of residuals was verified using the Shapiro–Wilk test, and the homogeneity of variances was assessed with Levene’s test. When assumptions were met, a one-way analysis of variance (ANOVA) was performed to determine significant differences among treatments, followed by Tukey’s post hoc test for pairwise comparisons. The significance level was set at *p* < 0.05.

For the determination of the effective concentration (EC_50_), lowest observed effect concentration (LOEC), and no observed effect concentration (NOEC), Probit analysis and ANOVA were applied. Growth factor (GF) and specific growth rate (µ) were calculated according to OECD TG 201 (2011), and the coefficient of variation (CV%) was computed across replicates to assess variability.

Relative quantification of photosynthetic gene expression was performed using a modified standard curve method as described by Steinberg et al. (2012) [66]. For each target gene (*atpB*, *psaA*, *psaB*, *psaD*, *psbA*, and *rbcL*) and the reference gene *18S* rRNA, standard curves were generated from serial dilutions of cDNA to determine slope, coefficient of determination (R^2^), and amplification efficiency (Eff%). When amplification efficiencies deviated from the ideal 90–110% range, efficiency values were explicitly incorporated into the expression calculations. In each experimental condition, expression of the target genes was normalized to *18S* rRNA, used as a stable endogenous reference in this model. Relative expression was calculated according to the following equation:

(4) $$\mathrm{Relative}\;\mathrm{expression}=\frac{mRNA\;of\;target\;gene}{mRNA\;of\;18S}$$

All data are presented as mean ± standard deviation (SD) from three independent biological replicates, each measured in technical triplicate.

Statistical significance was indicated in figures and tables by asterisks (*p* < 0.05; one-way ANOVA, Dunnett’s post hoc test), and this convention was applied consistently across all graphs and tables.

## 5. Conclusions

This study demonstrates that environmentally relevant concentrations of TiO_2_ nanoparticles elicit complex, gene-specific responses in *C. vulgaris*, which cannot be fully captured by conventional growth assays alone. While cell counts revealed a hormetic pattern with growth stimulation at intermediate exposures, molecular data showed that key photosynthetic genes were differentially regulated: *rbcL* was repressed at low doses, while *psaA* and *psaD* were induced at intermediate to high concentrations. These divergent trajectories highlight the dual nature of nanoparticle effects, where apparent stimulation at the cellular level coexists with underlying transcriptional stress.

From a risk assessment perspective, these findings emphasize the need to integrate classical endpoints with molecular biomarkers. Reliance on growth-based measurements alone could lead to contradictory or misleading interpretations, underestimating the potential for subtle disruptions in primary productivity. By incorporating gene expression profiles into environmental risk frameworks, a more sensitive and mechanistic evaluation of nanoparticle impacts can be achieved, improving our ability to predict ecological consequences.

Overall, the results indicate that TiO_2_ nanoparticles can induce hormetic responses in microalgae through an interplay between early repression of carbon fixation and compensatory induction of PSI components. Such molecular plasticity provides short-term resilience, but its sustainability under prolonged or community-level exposure remains uncertain. Future studies should therefore combine long-term bioassays with multi-level biomarkers to refine the ecological risk assessment of engineered nanoparticles in aquatic ecosystems.

## Figures and Tables

**Table 1:** Results of Dunnett’s T3 post hoc test comparing cell densities between *Chlorella vulgaris* exposed to different TiO_2_ nanoparticle concentrations and the control group after 72 h of exposure.

(I) Group	(J) Treatment	Mean Difference (I–J)	Standard Error	Sig.	95% Confidence Interval	
					Lower Bound	Upper Bound
Control	1.1 µg/L	5.6667	2.9059	0.633	−15.3685	26.7018
	4.4 µg/L	−24.6667	8.2731	0.372	−98.8309	49.4976
	8.8 µg/L	−28.3333	4.5216	0.09	−65.8700	9.2033
	17.6 µg/L	−61.000 *	2.0000	<0.001	−72.9276	−49.0724

* The difference in means is statistically significant at the 0.05 level.

**Table 2:** Efficiency and linearity parameters of primer quantification by qPCR for *Chlorella vulgaris*.

Gen	Slope	R^2^	Y-Inter	Eff%	Error
*18S*	−3.448	0.997	18.29	95.004	0.051
*atpB*	−3.2	0.981	23.149	105.341	0.113
*rbcL*	−3.294	0.983	20.448	101.194	0.107
*psaA*	−2.951	0.944	25.448	118.227	0.179
*psaB*	−3.375	0.985	26.552	97.832	0.106
*psaD*	−2.912	0.758	29.714	120.531	0.412
*psbA*	−3.458	0.983	20.29	94.637	0.114

**Table 3:** Levene’s test for homogeneity of variances and one-way ANOVA results for photosynthesis-related genes in *C. vulgaris* exposed to TiO_2_ nanoparticles. Significant values are indicated with an asterisk (*p* < 0.05).

Test of Homogeneity of Variances					
Dependent Variable	Levene Statistic	df1	df2	Sig.	
*atpB*	2.433	4	10	0.116	
*rbcL*	0.912	4	10	0.493	
*psaA*	3.625 *	4	10	0.045	
*psaB*	7.192 *	4	10	0.005	
*psaD*	6.332 *	4	10	0.008	
*psbA*	2.238	4	10	0.138	
ANOVA					
Dependent Variable	Sum of Squares	df1	Mean Square	F	Sig.
*atpB*	15.913	4	3.978	8.300	0.003 *
*rbcL*	1.853	4	0.463	14.568	0.000 *
*psaA*	882.128	4	220.532	8.992	0.002 *
*psaB*	10.981	4	2.745	7.092	0.006 *
*psaD*	615.215	4	153.804	13.031	0.001 *
*psbA*	1.901	4	0.077	0.484	0.748

**Table 4:** List of reference genes designed for the study of gene expression in *Chlorella v*.

Gene Name	GenBank Accession or Source	Forward Prime (5′3′)	Reverse Prime (5′3′)	Tm	%GC	PCR Product
*18S*	♦ Designed in this study	AACGGCTACCACATCCAAGG	GTCCCACCCGAAATCCAACT	55	55	250 pb
*atpB*	EF113499.1	CCAATTCACCGTTCAGCACC	TTTCCCTACACCTGCACCAC	55	55	144 pb
*psaA*	♦ Designed in this study	AAATGCAGACGTTGGTGGTG	CGCTAAACTGCCGAGACCTA	55	50	256 pb
*psaB*	GQ423926.1	GCCCAATGGATTCAAGCAGC	AGAACCACGAGCGTCAAGAG	55	55	255 pb
*psaD*	MG596028.1	AGCGTGTATAACCCAGCTCG	AGTGTCCTCCTCATACGCCT	55	55	267 pb
*psbA*	KX066373.1	CGTTGCCGGTGTATTTGGTG	ACAACTGGCCAAGCAGCTAA	55	55	240 pb
*rbcL*	MK295221.1	GCACGCTGTAATTGACCGTC	TCAACAAGAGCTGGCATGTG	55	55	285 pb

♦ Designed in this study (based on consensus sequences in Geneious Prime^®^ 1 January 2023).

## Data Availability

Data is contained within the article.

## Appendix A

**Table A1:** Relative expression values (mean ± SD) of photosynthesis-related genes (atpB, psaA, psaB, psaD, psbA, and rbcL) in *Chlorella vulgaris* after 72 h exposure to TiO_2_ nanoparticles. The table includes *p*-values from one-way ANOVA and post hoc tests.

Post Hoc Tests—Bonferroni							
Dependent Variable	(I) Treatments	(J) Treatments	Mean Difference (I–J)	Std. Error	Sig	Lower Bound	Upper Bound
*atpB*	Control	1.1 µg/L	1.906	0.144	0.071	−0.118	3.9308
		4.4 µg/L	1.722	0.589	0.123	−0.302	3.7464
		8.8 µg/L	−0.788	0.344	1.000	−2.812	1.2364
		17.6 µg/L	1.065	0.276	0.889	−0.959	3.0894
*rbcL*	Control	1.1 µg/L	0.823 *	0.169	0.002	0.301	1.3441
		4.4 µg/L	0.897 *	0.118	0.001	0.375	1.4181
		8.8 µg/L	0.170	0.188	1.000	−0.351	0.6918
		17.6 µg/L	0.503	0.139	0.062	−0.019	10.244
*psaA*	Control	1.1 µg/L	4.627	0.479	1.000	−9.854	19.1084
		4.4 µg/L	2.575	5.293	1.000	−11.907	17.0561
		8.8 µg/L	−17.020 *	7.830	0.018	−31.501	−2.5383
		17.6 µg/L	1.718	3.920	1.000	−16.199	12.7634
*psaB*	Control	1.1 µg/L	1.317	0.133	0.268	−0.502	3.1367
		4.4 µg/L	1.705	0.411	0.073	−0.115	3.5240
		8.8 µg/L	−0.614	0.238	1.000	−2.433	1.2057
		17.6 µg/L	0.942	0.273	0.934	−0.877	2.7613
*psaD*	Control	1.1 µg/L	5.570	0.959	0.752	−4.476	15.6157
		4.4 µg/L	6.567	0.872	0.413	−3.479	16.6127
		8.8 µg/L	−11.375 *	6.772	0.023	−21.421	−1.3293
		17.6 µg/L	1.638	2.399	1.000	−8.408	11.6837
*psbA*	Control	1.1 µg/L	0.215	0.567	1.000	−0.952	1.3817
		4.4 µg/L	0.358	0.505	1.000	−0.809	1.5247
		8.8 µg/L	0.292	0.204	1.000	−0.875	1.4594
		17.6 µg/L	0.022	0.202	1.000	−1.145	1.1890

* The difference in means is statistically significant at the 0.05 level.
